# Passive and post-exercise cold-water immersion augments PGC-1α and VEGF expression in human skeletal muscle

**DOI:** 10.1007/s00421-016-3480-1

**Published:** 2016-10-03

**Authors:** C. H. Joo, R. Allan, B. Drust, G. L. Close, T. S. Jeong, J. D. Bartlett, C. Mawhinney, J. Louhelainen, J. P. Morton, Warren Gregson

**Affiliations:** 1 Football Exchange, Research Institute for Sport and Exercise Sciences, Liverpool John Moores University, Tom Reilly Building, Byrom St Campus, Liverpool, L3 3AF UK; 2 Honam University, Gwangsan-gu, Gwangju, South Korea; 3 Institute of Sport, Exercise and Active Living, College of Sport and Exercise Sciences, Victoria University, Melbourne, Australia; 4 Faculty of Pharmacy and Biomolecular Sciences, Liverpool John Moores University, Byrom St Campus, Liverpool, UK

**Keywords:** PGC-1α, VEGF, CWI

## Abstract

**Purpose:**

We tested the hypothesis that both post-exercise and passive cold water immersion (CWI) increases PGC-1α and VEGF mRNA expression in human skeletal muscle.

**Method:**

*Study 1* Nine males completed an intermittent running protocol (8 × 3-min bouts at 90 % $\dot{V}{\text{O}}_{2} \hbox{max}$, interspersed with 3-min active recovery (1.5-min at 25 % and 1.5-min at 50 % $\dot{V}{\text{O}}_{2} \hbox{max}$) before undergoing CWI (10 min at 8 °C) or seated rest (CONT) in a counterbalanced, randomised manner. *Study 2* Ten males underwent an identical CWI protocol under passive conditions.

**Results:**

*Study 1* PGC-1α mRNA increased in CONT (~3.4-fold; *P* < 0.001) and CWI (~5.9-fold; *P* < 0.001) at 3 h post-exercise with a greater increase observed in CWI (*P* < 0.001). VEGFtotal mRNA increased after CWI only (~2.4-fold) compared with CONT (~1.1-fold) at 3 h post-exercise (*P* < 0.01). *Study 2* Following CWI, PGC-1α mRNA expression was significantly increased ~1.3-fold (*P* = 0.001) and 1.4-fold (*P* = 0.0004) at 3 and 6 h, respectively. Similarly, VEGF165 mRNA was significantly increased in CWI ~1.9-fold (*P* = 0.03) and 2.2-fold (*P* = 0.009) at 3 and 6 h post-immersion.

**Conclusions:**

Data confirm post-exercise CWI augments the acute exercise-induced expression of PGC-1α mRNA in human skeletal muscle compared to exercise per se. Additionally CWI per se mediates the activation of PGC-1α and VEGF mRNA expression in human skeletal muscle. Cold water may therefore enhance the adaptive response to acute exercise.

## Introduction

As a transcriptional co-activator, peroxisome proliferator-activated receptor γ coactivator-1α (PGC-1α) is now well accepted as a critical regulator of mitochondrial biogenesis in skeletal muscle (Puigserver and Spiegelman 2003). The functional importance of this proposed “master regulator” is also recognised by observations from rodent studies demonstrating that overexpression improves insulin sensitivity (Handschin and Spiegleman 2006; Lira et al. 2010), protects against sarcopenia (Ji and Kang 2015) and improves exercise capacity (Baar et al. 2002; Lin et al. 2002). Given the relevance of the aforementioned physiological adaptations for human health and physical performance, the factors affecting the regulation of PGC-1α expression in human skeletal muscle is now an intensive area of research. In this regard, it is noteworthy that acute exercise induces PGC-1α expression (Perry et al. 2010; Bartlett et al. 2012, 2013) in a manner that is intensity dependent (Egan et al. 2010), mediated by upstream signalling through key cell signalling kinases such as AMPK and p38MAPK.

Consistent with its initial discovery as “cold-inducible” (Puigserver et al. 1998), there is now a growing body of literature from both rodent (Oliveira et al. 2004; Kim et al. 2005; Stancic et al. 2013) and human tissues (Slivka et al. 2012, 2013; Ihsan et al. 2014, 2015) demonstrating that exposure to an acute and prolonged “cold” stimulus (as mediated via cold ambient temperatures or cold water immersion) also up-regulates PGC-1α expression. When taken together, these data therefore suggest that acute cold exposure may enhance the response over and above the stress of exercise alone to induce the classical hallmark adaptations to endurance training. Indeed, Ihsan et al. (2014) recently observed that post-exercise cold-water immersion (CWI) augmented skeletal muscle PGC-1α mRNA expression when compared to a non-immersed limb that completed an identical work-load during exercise. Furthermore, the same group demonstrated that regular post-exercise CWI application during 4 weeks of endurance training up-regulated PGC-1α protein content, along with several mitochondrial proteins thus suggesting that regular CWI may enhance training-induced mitochondrial biogenesis (Ihsan et al. 2015). It should be noted, however, that Ihsan et al. (2014) observed that exercise alone did not induce PGC-1α mRNA expression in the non-immersed limb (despite exercise being a potent stimulus to induce PGC-1α expression), thereby suggesting that this response was mediated entirely by cold-induced mechanisms such as increased β-adrenergic activity (Hensel and Boman 1960). Consequently, a fundamental question remains as to the relative importance of the cold stimulus upon mediating both mitochondrial and angiogenic pathways and/or whether superimposing the stress of cooling on the prior stress of exercise represents a superior stimulus.

PGC-1α also plays an important regulatory role in mediating exercise-induced angiogenesis through its effects on vascular endothelial growth factor (VEGF) (Chinsomboon et al. 2009), thereby resulting in enhanced capillary density and improved conduit and microvascular function (Hoier and Hellsten 2014). Chronic exposure to a cold environment has been reported to increase capillary density in animals (Sillau et al. 1980; Suzuki et al. 1997) and human skeletal muscle (Bae et al. 2003) and is thought to be mediated through the regulatory role of PGC-1α in facilitating the key pro-angiogenic factor in skeletal muscle, VEGF (Chinsonboom et al. 2009). To date, limited attention has focused upon the influence of acute CWI on VEGF expression. In animals, Kim et al. (2005) demonstrated that prolonged CWI (1 h day^−1^, 5 days week^−1^ for 20 weeks) evoked higher expressions of VEGF_165_ mRNA and protein when compared to a control. In contrast, in humans, Ihsan et al. (2014), failed to observe any effect of CWI on VEGF mRNA despite apparent activation of PGC-1α.

With this in mind, the aims of the present study were twofold. In Study 1, we aimed to test the hypothesis that post-exercise CWI increases both PGC-1α and VEGF mRNA expression in human skeletal muscle when compared to exercise alone. To this end, we adopted a repeated measures design whereby male subjects completed an acute bout of high-intensity interval running (previously shown in our laboratory to up-regulate PGC-1α mRNA expression, Bartlett et al. 2012) followed by a 10-min 2-legged CWI protocol that is common practice for athletic populations. In Study 2, we subsequently tested the hypothesis that CWI without the prior stress of exercise was sufficient to also mediate the activation of such molecular pathways.

## Methods

### Ethical approval

All subjects gave written informed consent to participate after details and procedures of the study had been fully explained. All subjects had no history of neurological disease or musculoskeletal abnormality and none was under any pharmacological treatment during the course of the study. All procedures performed in the study were approved by the University Ethics Committee and in accordance with the 1964 Helsinki declaration and its later amendments.

### Study overview

Study 1 examined the impact of post exercise two-legged CWI on these markers of mitochondrial biogenesis and angiogenesis. Following baseline measures, subjects completed an intermittent running protocol followed by CWI or a control condition (seated rest; CONT). Subsequent to this, subjects recovered in a semi-reclined position under normal laboratory temperatures (23 ± 0.5 °C) until 3 h post-exercise. Study 2 examined the impact of passive CWI on PGC-1α and VEGF mRNA expression and protein content. Subjects arrived at the laboratory and underwent a series of resting measures before completing a CWI protocol without prior exercise. Subjects then remained in a semi-reclined position until 6 h had passed whilst various physiological measures were conducted and blood and muscle biopsies were sampled. All trials were conducted under normal laboratory ambient temperatures (23 ± 0.5 °C) and at the same time of day (~9 am start) in order to avoid the circadian variation in internal body temperature. Trials were randomly counterbalanced with at least 1 week between conditions.

### Baseline assessments

Roughly 10–14 days prior to the subjects scheduled test day they underwent a standard incremental treadmill test for determination of maximal oxygen uptake ($\dot{V}{\text{O}}_{2} \hbox{max}$). This was completed in Study 1 in order to permit calculation of running speeds required during the intermittent exercise protocol and in Study 2 for population purposes. Briefly, the protocol commenced at a treadmill speed of 10 km h^−1^ for 4-min followed by 2-min stages at 12, 14 and 16 km.h^−1^, respectively. Upon completion of the 16 km h^−1^ stage the treadmill incline was increased by 2 % every 2-min thereafter until volitional exhaustion. The $\dot{V}{\text{O}}_{2} \hbox{max}$ was only achieved by reaching the following end point criteria: (1) heart rate (HR) within 10 beats min^−1^ of age-predicted maximum, (2) respiratory exchange ratio (RER) >1.1, and (3) plateau of oxygen uptake ($\dot{V}{\text{O}}_{2}$) despite increased workload.

Test day preparation and baseline measurements were similar for both studies. On the morning of testing subjects arrived at the laboratory following an overnight fast having refrained from exercise, alcohol, tobacco and caffeine for 72 h. All subjects recorded nutritional and fluid intake prior to the first exercise trial to permit them to repeat their preparation for the remaining trial. Subjects were instructed to ingest 5 ml of water per kg of body mass 2 h before arriving at the laboratory. Nude body mass (KG; Seca, Birmingham, UK) was measured before subjects rested for 30 min in a semi-reclined position. Baseline measures of HR (Polar RS400, Polar Electro, Kempele, Finland), $\dot{V}{\text{O}}_{2}$ (Jaeger Oxycon Pro, Carefusion, Leibnizstrasse, Germany), and skin and rectal temperature (CTF 9004, Ellab) were taken at this point.

#### Study 1

*Experimental design* Nine healthy active males volunteered to participate in the study (mean ± SD: age 25 ± 4 years, height 174.7 ± 4.7 cm, body mass 78.3 ± 9.2 kg and $\dot{V}{\text{O}}_{2} \hbox{max}$ were 58.1 ± 7.2 ml kg^−1^ min^−1^). Following baseline assessments, subjects completed 60-min high intensity intermittent exercise on a motorised treadmill (HP Cosmos, Pulsar, Germany). The intermittent running protocol consisted of a 10-min warm up at a running velocity corresponding to 70 % of $\dot{V}{\text{O}}_{2} \hbox{max}$. This was followed by eight 3-min bouts at a running velocity corresponding to 90 % $\dot{V}{\text{O}}_{2} \hbox{max}$ interspersed with 3-min active recovery periods (1.5-min at a velocity corresponding to 25 % $\dot{V}{\text{O}}_{2} \hbox{max}$ followed by 1.5-min at velocity corresponding to 50 % $\dot{V}{\text{O}}_{2} \hbox{max}$). A 5-min cool-down period (50 % of $\dot{V}{\text{O}}_{2} \hbox{max}$) was undertaken following completion of the final exercise bout. This protocol mediated limited structural and functional muscle damage in the subjects and has previously been shown to up-regulate signalling responses of molecular adaptation (Joo 2015; Bartlett et al. 2012).

Immediately after the intermittent running protocol each subject was required to complete CWI or remain seated under normal laboratory temperatures. CWI consisted of subjects completing a 10-min period of immersion in 8 °C water (2 × 5 min separated by a 2 min intermission above the water). Subjects were lowered and raised into and from the water to the iliac crest using an electronic hoist system. Subsequently subjects remained seated in the water-immersion lab, in a semi-reclined position, where subjective ratings of perceived shivering (Visual analogue scale; 1- No Shivering, 5-Severe Shivering), heart rate, $\dot{V}{\text{O}}_{2}$, rectal and skin temperature (thigh and calf) were measured continuously during CWI and recovery. Subjects remained in a semi-reclined seated position in the water-immersion lab until 1-h post-exercise. Subsequently, subjects rested on a bed for a further 2 h after changing from wet clothes. Ratings of perceived exertion (RPE) were obtained immediately following each exercise bout. Heart rate, $\dot{V}{\text{O}}_{2}$, shivering, rectal and skin temperature (thigh and calf) were measured continuously during CWI and recovery for 30-min. Muscle temperature was measured post-exercise, post-CWI and at 1 and 3 h post-exercise. Muscle biopsies were obtained at pre-exercise, immediately post-exercise and 3 h post-exercise. Venous blood samples were also obtained at pre-exercise, immediately post-exercise and 1 and 3 h post-exercise.

#### Study 2

*Experimental design* Ten healthy active men volunteered to participate in the study (mean ± SD: age 24 ± 1 year, height 175.3 ± 4 cm, body mass 79.5 ± 6 kg and $\dot{V}{\text{O}}_{2} \hbox{max}$ were 55.5 ± 7.3 ml kg^−1^ min^−1^). Following completion of the baseline assessments each subject was required to complete CWI (as outlined in Study 1) or remain seated for 10-min under normal laboratory temperatures (CONT). Subjects were then allowed to change to dry clothing before resting in a semi-reclined position on a bed for a further 3 h. Subjects were fed after +3 h measurements in the form of a sandwich plus low calorie flavoured water (150 kcal; 21 g CHO, 11 g protein, 2 g fat). Muscle temperature was measured immediately post-immersion and 0.5, 1.5, 3, and 6 h post-immersion. Venous blood samples were also taken at these time points. Muscle biopsies were obtained pre-CWI, at 3 and 6 h post-immersion.

### Thermoregulatory measurements

Muscle temperature was assessed using a technique previously described (Mawhinney et al. 2013). Briefly, a needle thermistor (13050; Ellab, Rodovre, Denmark) was inserted into the vastus lateralis. Thigh skinfold thickness was measured using Harpenden skinfold callipers (HSK BI; Baty International, West Sussex, UK) and divided by 2 to determine the thickness of the thigh subcutaneous fat layer over each participant’s vastus lateralis. The needle thermistor was then placed at a depth of 3 cm, plus one-half of the skinfold measurement, for the determination of deep muscle temperature. After stabilisation of temperature the thermistor was withdrawn at increments of 1 cm for the determination of muscle temperature at 2 and 1 cm below the subcutaneous layer. Core temperature was assessed via a rectal thermistor (MRV-55044-A, Ellab, Roedovre, Denmark) self-inserted 10–15 cm beyond the anal sphincter. Skin temperature probes were attached to the lateral upper thigh and medial calf (MHF-18050-A, Ellab) using adhesive surgical tape.

### Blood analyses

Venous blood samples were drawn from a superficial vein in the anti-cubital crease of the forearm using standard venepuncture techniques (Vacuatiner Systems, Becton, Dickinson, Europe). Samples were collected into vacutainers (Becton, Dickinson) containing EDTA or serum separation tubes and stored on ice or at room temperature (serum samples, ~1 h) until centrifugation at 1500 rev min^−1^ for 15-min at 4 °C. Following centrifugation, aliquots of plasma and serum were stored at −80 °C for later analysis. Samples from both studies were analysed for blood glucose and lactate using commercially available kits (Randox Laboratories, Antrim, UK). Plasma adrenaline and noradrenaline concentrations (Study 2 only) were measured using liquid chromatography-tandem mass spectrometry (Peaston et al. 2010). All samples were analysed in duplicate.

### Muscle biopsies

Muscle biopsies were obtained from the vastus lateralis muscle under local anaesthesia (0.5 % marcaine) using a Pro-Mag 2.2 biopsy gun (MD-TECH, Manan Medical Products, Northbrook, IL, USA) as previously described (Morton et al. 2009). Each incision was anaesthetised individually and occurred at a distance of 2–3 cm from the previous incision. Once obtained, samples (~50 mg of tissue) were immediately frozen in liquid nitrogen and stored at −80 °C for later analysis. The biopsied limb was counterbalanced for leg dominance between trials, in both studies. The incision was protected using butterfly closure sterile strips and waterproof adhesive dressings.

### Muscle analysis

*Real-time RT-PCR* Total RNA was isolated from muscle biopsies (20–30 mg) using Trizol reagent (Invitrogen), according to the manufacturer’s protocol. RNA quality and quantity were determined using Implen Nanophotometer (Implen, Munchen, Germany) and the RNA was stored at −80 °C. cDNA was synthesised using random hexamers (Applied Biosystems) and Superscript III enzyme (Invitrogen), using manufacturer’s protocol. Gene specific expression data was obtained using probes selected from Human Universal Probe Library (Roche Diagnostics) with compatible oligonucleotide primers (MWG Eurofins), except for VEGF_165_ for which Green technology was applied. One microliter of each cDNA sample was analysed in triplicate with negative controls using AB 7500 Real-Time Quantitative PCR instrument (Applied Biosystems) and Agilent Brilliant II qPCR Master Mix with Low ROX (Agilent Technologies). One microliter of cDNA, 500 nM of primer and 200 nM of probe were used for each 20-μl reaction. Primers were designed to detect for PGC-1α (Forward: CAAGCCAAACCAACAACTTTATCTCT, Reverse: ACGACCAAATCCGTTGACTC; Probe 60), VEGF (Forward: CCT TGCTGCTCTACCTCCAC, Reverse: CCACTTCGTGATGATTCTGC; Probe 29), VEGF_165_ (Forward: TGTGAATGCAGACCAAAGAAAGA, Reverse: TGCTTTCTCCGCTCTGAGC) and GAPDH (Forward: GCTCTCTGCTCCTCCTGTTC, Reverse: ACGACCAAATCCGTTGACTC; Probe 60). The following cycling parameters were used: 50 °C for 2 min, initial denaturation at 95 °C for 10 min, followed by 40 cycles of denaturation at 95 °C for 15 s and annealing/elongation at 60 °C for 1 min. For SYBR green reactions additional post-qPCR melting curve analysis was also performed for QC-purposes. Data was collected and analysed using AB SDS 1.43 Software (Applied Biosystems, Foster City, USA). Changes in mRNA content were calculated according to the 2-ΔΔCt method where GAPDH was used as the housekeeping gene. In order determine the optimum housekeeping gene(s) GAPDH, β2M and β-actin pre-tests were run on all samples to ensure no variability between time points. The use of GAPDH as a single reference gene was used due to variability in other housekeeping genes (β2M and β-actin).

*Western blotting* Approximately 20–30 mg of frozen muscle was ground to powder and homogenised in 120 µl of ice cold lysis buffer [25 mM Tris/HCl (pH 7.4), 50 mM NaF, 100 mM NaCl, 5 mM EGTA, 1 mM EDTA, 10 mM Na-Pyrophosphatase, 1 mM Na_3_VO_4_, 0.27 M sucrose, 1 % Triton X-100, 0.1 % 2-mercaptoethanol] and supplemented with a protease inhibitor tablet (Complete mini, Roche Applied Science, West Sussex, UK). Homogenates were centrifuged at 14,000*g* for 10-min at 4 °C and the supernatant was collected. The protein content of the supernatant was determined using a bicinchoninic acid assay (Sigma, UK). Each sample was diluted with an equal volume of 2X Laemmli buffer (National Diagnostics, USA) and boiled for 5-min at 100 °C. For each blot, a standard and internal control was loaded along with 50 µg of protein from each sample and then separated in Tris–glycine running buffer (10 X Tris/Glycine, Geneflow Ltd, Staffordshire, UK) using self-cast 4 % stacking and 10 % separating gels (National Diagnostics, USA). Gels were transferred semi-dry onto nitrocellulose membrane (Geneflow Ltd, Staffordshire, UK) for 2 h at 200 V and 45 mA per gel in transfer buffers (anode 1; 0.3 M Tris, 20 % methanol, pH 10.4; anode 2; 0.25 M Tris, 20 % methanol, pH 10.4; cathode; 0.4 M 6-amino hexanoic acid, 20 % methanol, pH 7.6). After transfer, membranes were blocked for 1 h at room temperature in Tris-buffered saline (TBST: 0.19 M Tris pH 7.6, 1.3 M NaCl, 0.1 %Tween-20) with 5 % non-fat milk. The membranes were then washed for 3 × 5-min in TBST before being incubated overnight at 4 °C with specific protein total content antibodies; GAPDH (Cell Signalling, UK), VEGF (Santa Cruz Biotechnology, Germany) and PGC-1α (Calbiochem, Merck Chemicals, UK) all at concentrations of 1:1000 in 1 X TBST. The next morning, membranes were washed for a further 3 × 5-min in TBST and subsequently incubated with anti-species horseradish peroxidise-conjugated secondary antibody (Bio-Rad, UK or Dako, UK) for 1 h at room temperature. After a further 3 × 5-min washes in TBST, membranes were exposed in a chemiluminescence liquid (SuperSignal, Thermo Fisher Scientific, Rockford, IL, USA) for 5-min. Membranes were visualised using a Bio-Rad Chemi-doc system, and band densities were determined using ImageLab image-analysis software. In order to ensure the antibodies used in each study were specific to the protein of interest, a secondary control was run on every gel.

### Statistical analysis

#### Study 1

All data are presented as mean ± SD. A two-factor (condition × time) within-subjects general linear model (GLM) was undertaken to determine any treatment differences between the CONT and COLD conditions. The assumption of sphericity (homogeneity of covariance) was assessed and corrected for using the Huynh–Feldt epsilon. Because there were only two levels in the main effect of condition, follow-up multiple comparisons were not necessary. A significant effect of time was followed up with planned multiple contrasts in line with the a priori hypotheses. Therefore, data at the specific time points were compared with the baseline (first) time point using Newman–Keuls multiple contrasts. Where a significant interaction between condition and time was observed, differences between conditions were examined at each time point using Newman–Keuls multiple contrasts. Baseline values were compared using a paired samples *t* test. One sample t test was used to compare the immersion-induced change in shivering and changes of muscle temperature between baseline and other time points. The alpha level for evaluation of statistical significance was set at *P* < 0.05.

*Study 2* All data are presented as mean ± SD. Any systematic changes in thermoregulatory responses, HR, $\dot{V}{\text{O}}_{2}$, mRNAs, proteins and blood variables during resting period were assessed using one-way within-subjects GLM. Post-hoc analysis by Newman–Keuls test was undertaken to examine which trials were significantly different from pre-immersion. One sample t test was used to compare the immersion-induced change in shivering and changes of muscle temperature between baseline and other time points. The alpha level for evaluation of statistical significance was set at *P* < 0.05.

## Results

### Study 1

Physiological responses during exercise and recovery are presented in Table 1. Exercise HR and RPE were similar between CONT and CWI conditions (*P* > 0.05). Heart rate significantly increased from the first to final bout under both conditions (CONT, 176 ± 8–183 ± 9 beats min^−1^; CWI, 179 ± 6–183 ± 7 beats min^−1^; *P* < 0.001), with the final exercise bout equating to 94 and 95 % of HR_max_ in the CONT and CWI conditions respectively. Rating of perceived exertion following the final bout was 19 ± 1 and 20 ± 1 in the CONT and CWI condition respectively. Heart rate during CWI showed a significant increase (*P* < 0.05) in the first 2 min of immersion before returning to baseline values. $\dot{V}{\text{O}}_{2}$ increased above pre-immersion levels in the 2nd and 6th minute of CWI (*P* < 0.05), whilst values in CONT remained stable throughout. Additionally, subjective measures of shivering showed significant increases in CWI (2nd and 8th minutes only; *P* < 0.05) with subjects reporting slight shivering. No differences were detected elsewhere (*P* > 0.05; Table 1). Blood glucose and lactate concentrations were similar between conditions pre-exercise, increasing in both CONT and CWI immediately after exercise (*P* < 0.05; except for glucose CONT post *P* = 0.07). Decreases were seen 1 h post-exercise in both conditions (*P* > 0.05) and remained unchanged at 3 h post exercise (Table 2).

**Table 1:** Physiological and metabolic variables pre-, during and post-immersion

Study	Condition	Pre-immersion	2	4	6	8	10	12	+10 min	+20 min	+30 min
HR (beats min^−1^)											
1	CON	89 ± 10	87 ± 11	89 ± 10	88 ± 10	87 ± 9	87 ± 10	90 ± 12	83 ± 10	82 ± 11	84 ± 11
	CWI	90 ± 8	102 ± 6*^+^	96 ± 5	91 ± 8	92 ± 8	90 ± 7	89 ± 8	83 ± 6	82 ± 9	81 ± 5
2	Passive	67 ± 9	80 ± 14*	67 ± 9	65 ± 9	63 ± 9	62 ± 9	60 ± 5	55 ± 9	54 ± 7	57 ± 8
$\dot{V}{\text{O}}_{2}$ (ml kg^−1^ min^−1^)											
1	CON	6.68 ± 1.05	6.55 ± 1.17	6.90 ± 1.52	6.59 ± 1.15	6.56 ± 1.29	6.63 ± 0.90	6.99 ± 1.83	6.28 ± 1.22	6.42 ± 1.35	6.32 ± 0.87
	CWI	7.36 ± 1.11	9.77 ± 1.58*	8.13 ± 1.90	8.71 ± 2.51*	7.19 ± 0.98	7.94 ± 1.14	8.22 ± 1.04	7.41 ± 2.07	6.79 ± 0.75	6.95 ± 1.27
2	Passive	5.47 ± 1.39	6.96 ± 0.65	6.52 ± 1.49	7.34 ± 2.01*	6.92 ± 2.64*	7.05 ± 2.86	7.14 ± 2.11	4.65 ± 1.63	3.93 ± 0.94*	4.34 ± 1.31
Shivering (AU)											
1	CON	1 ± 0	1 ± 0	1 ± 0	1 ± 0	1 ± 0	1 ± 0	1 ± 0	1 ± 0	1 ± 0	1 ± 0
	CWI	1 ± 0	2 ± 0.63*^+^	1.67 ± 0.81	1.33 ± 0.52	1.83 ± 0.75*^+^	1.83 ± 0.98	2 ± 1.26	1.5 ± 0.84	1.5 ± 0.55	1.5 ± 0.55
2	Passive	1 ± 0	2 ± 0.94*	2 ± 1.05*	1.7 ± 0.67*	1.8 ± 0.63*	1.7 ± 0.67*	1.7 ± 0.67*	1.2 ± 0.42	1 ± 0	1 ± 0

Values are mean ± SD; *n* = 9 subjects (Study 1) *n* = 10 subjects (Study 2)

*PASS* passive cold water immersion, *CON* post-exercise control trial, *CWI* post-exercise CWI trial

* *P* < 0.05 versus pre-immersion value; ^+^ *P* < 0.05 versus CON

**Table 2:** Study 1 Plasma glucose and lactate before, immediately after exercise, 1 h and 3 h post-exercise in the Cont and CWI condition (*n* = 9, mean ± SD)

	CON				CWI			
	Pre	Post	1 h	3 h	Pre	Post	1 h	3 h
Glucose, mmol/l	4.0 ± 0.9	5.1 ± 1.4	4.4 ± 0.6	4.5 ± 0.4	4.0 ± 0.5	5.4 ± 1.5*	4.6 ± 0.6	4.4 ± 0.5
Lactate, mmol/l	2.2 ± 0.6	7.4 ± 3.3*	3.3 ± 1.2	2.7 ± 0.7	2.4 ± 0.5	7.7 ± 3.0*	3.4 ± 1.5	3.0 ± 0.6

Values are mean ± SD. *n* = 9 subjects (Study 1)

*CON* post-exercise control condition, *CWI* post-exercise CWI condition

* *P* < 0.05 versus pre-exercise value

**Fig. 1:**
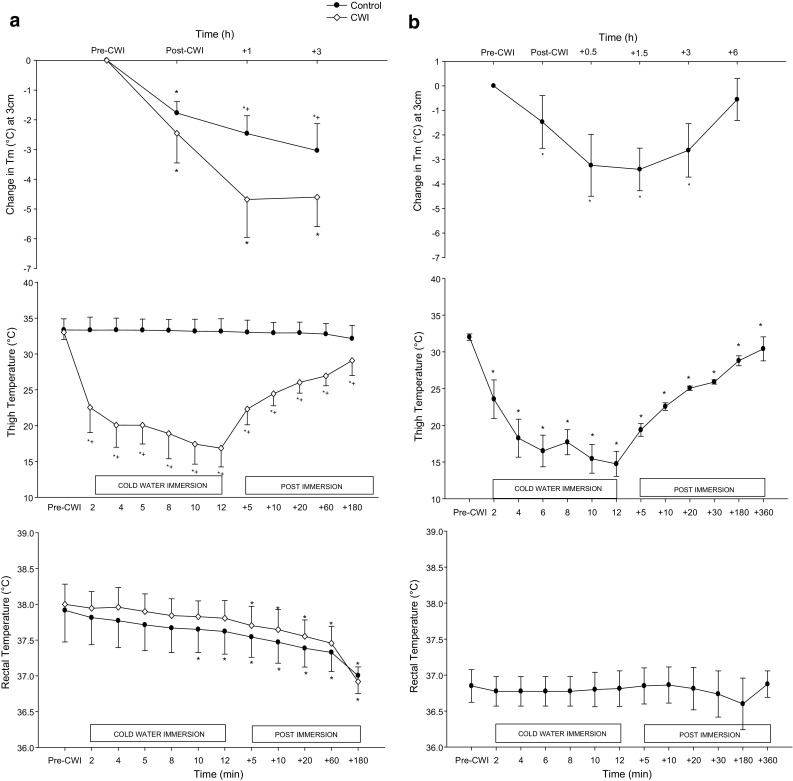
Changes in muscle temperature (Tm) (3 cm), thigh temperature and rectal temperature post-exercise (Study 1; **a**) and Passive CWI (Study 2; **b**). **a** *Significant difference from pre-immersion (*P* < 0.05). ^+^Significant difference between conditions (*P* < 0.05). **b** *P < 0.05 significantly different from pre-immersion

Rectal temperature was similar between conditions throughout the experimental protocol (*P* > 0.05). Rectal temperature declined throughout immersion and the post-immersion recovery period with the greatest decrement observed at 3 h post-exercise in both conditions (*P* < 0.001). Post exercise thigh and calf skin temperatures were similar between conditions. CWI induced significant declines (*P* < 0.05) in thigh and calf temperatures compared to CONT, with the largest reduction (~16 °C CWI vs. CONT) occurring at the end of CWI. CONT temperatures remained relatively stable throughout the immersion and post-immersion recovery periods whilst thigh and calf skin temperatures remained significantly lower in CWI throughout the 3 h post-exercise period (*P* < 0.05; Fig. 1; Tcalf data not shown). Muscle temperature (3 cm depth) was similar between conditions immediately post-immersion (*P* > 0.05). Muscle temperature decreased during the 3 h post exercise period in both conditions with greater decreases in CWI compared with CONT at 1 h and 3 h post-exercise decreases (*P* < 0.05; Fig. 1).

**Fig. 2:**
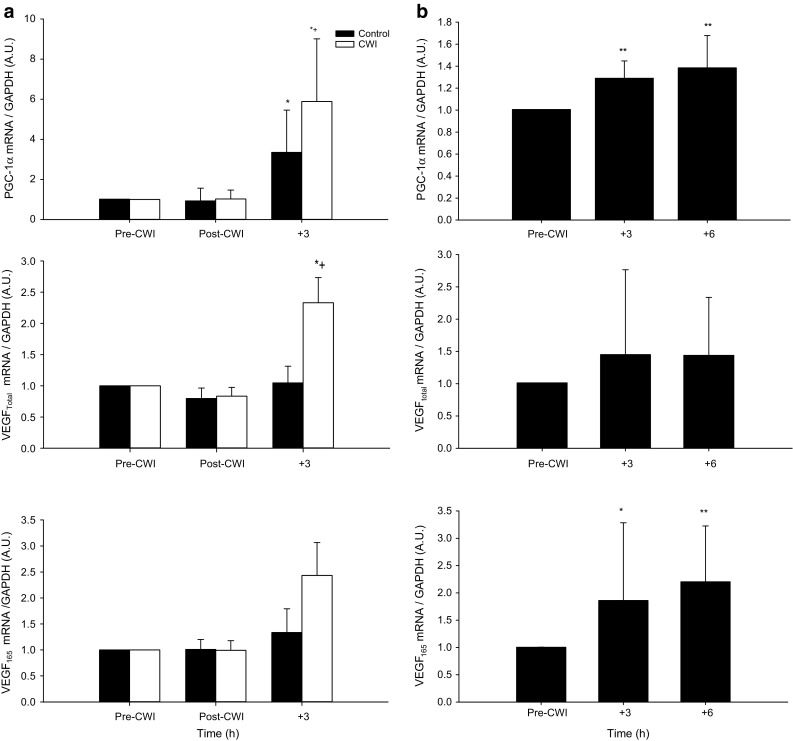
PGC-1α, VEGF_total_, VEGF_165_ mRNA responses to post-exercise (Study 1, **a**) and passive CWI (Study 2, **b**). **a** *Significant difference from pre-exercise (*P* < 0.05). ^+^Significant difference between conditions (*P* < 0.05). **b** *P < 0.05, **P < 0.01 significantly different from pre-immersion

**Fig. 3:**
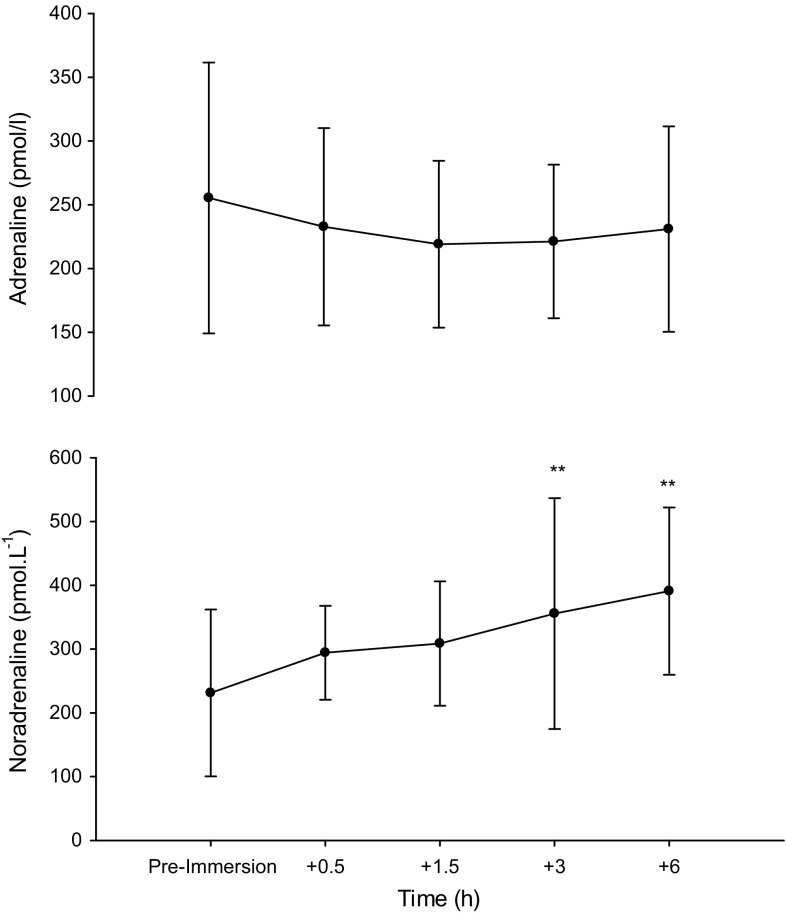
Catecholamine responses pre- and post-passive CWI. **P < 0.01; significantly different from pre-immersion

**Fig. 4:**
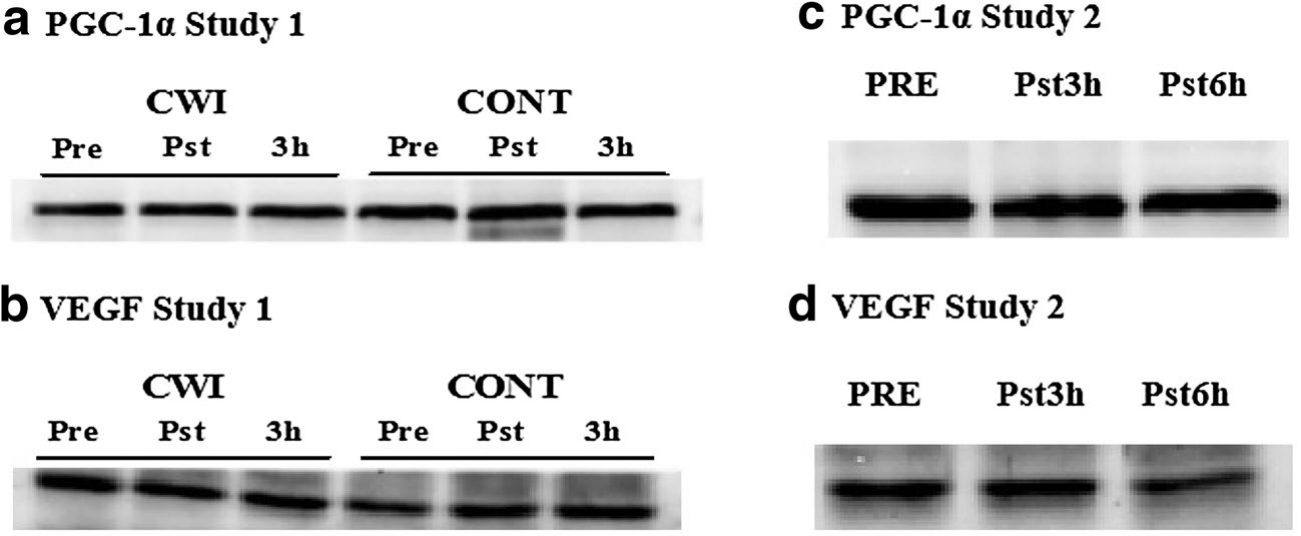
Representative Western Blots for PGC-1α and VEGF after post-exercise CWI (Study 1; **a**, **b**) and passive CWI (Study 2; **c**, **d**)

Post-exercise PGC-1α mRNA remained similar to pre-exercise under both conditions (*P* > 0.05). However, PGC-1α mRNA increased in both CONT (~3.4-fold; *P* < 0.001) and CWI (~5.9-fold; *P* < 0.001) at 3 h post-exercise with a greater increase observed in CWI (*P* < 0.001) (Fig. 2). No change in total protein content of PGC-1α when expressed relative to GAPDH was observed at any time point in CONT or CWI conditions (*P* > 0.05) (Fig. 4). VEGF_total_ mRNA did not change immediately after exercise in both conditions (*P* > 0.05). However, VEGF_total_ mRNA increased after CWI only (~2.4-fold) compared with CONT (~1.1-fold) at 3 h post-exercise (*P* < 0.01). Post exercise VEGF_165_ remained similar to pre-exercise under both conditions (*P* > 0.05). However, there was a trend for VEGF_165_ to increase after 3 h post-exercise after CWI (~2.5-fold) compared with CONT (~1.4-fold; *P* = 0.06) (Fig. 2). No change in total protein content of VEGF when expressed relative to GAPDH was observed at any time point in CONT or CWI conditions (*P* > 0.05) (Figs. 3, 4).

### Study 2

Passive CWI had no impact on rectal temperature, however, it was able to induce significant decreases in 3 cm muscle temperature immediately post immersion and up to 3 h post-exercise (*P* < 0.05). Skin temperature (thigh and calf) showed peak decreases of ~15 °C by the end of the immersion period, gradually warming throughout the recovery period yet always remaining lower than pre-immersion values (*P* < 0.05) (Fig. 1; Tcalf values not shown).

Consistent with study 1, HR showed an initial cold shock response with significant increases in the first 2-mins of CWI (*P* < 0.001) before returning, and remaining, at levels similar to pre-immersion. $\dot{V}{\text{O}}_{2}$ was elevated above resting values throughout CWI (significantly at 6 and 8 min; *P* = 0.03 and 0.04, respectively) before returning to pre-immersion values by 10 min. Despite a drop in $\dot{V}{\text{O}}_{2}$ below pre-immersion values at 20-mins post-immersion (*P* = 0.05) by 30-mins post-exercise $\dot{V}{\text{O}}_{2}$ had returned to baseline. Subjective measures of shivering indicated ‘slight shivering’ during the CWI protocol only (*P* = 0.01). No shivering was reported in the immediate recovery period (30 min) (*P* < 0.05) (Table 1). Blood glucose, lactate and plasma adrenaline were unchanged from baseline values throughout the immersion and recovery periods (*P* > 0.05). Plasma noradrenaline was significantly increased at 3 and 6 h post immersion (*P* < 0.01) (Fig. 3).

After CWI, PGC-1α mRNA content was significantly increased ~1.3-fold and 1.4-fold at 3 h (*P* = 0.001) and 6 h (*P* = 0.0004), respectively. No changes were observed in PGC-1α protein content (*P* = 0.25). Similarly, VEGF_165_ mRNA was significantly increased after CWI ~1.9-fold at 3 h (*P* = 0.03) and 2.2-fold at 6 h (*P* = 0.009) post-immersion (Fig. 2). However, there were no changes in VEGF_total_ mRNA (*P* = 0.33) and VEGF protein content (*P* = 0.97) during the post-immersion period. Representative Western Blots are shown in Fig. 4.

## Discussion

The aim of the present study was to initially test the hypothesis that post-exercise CWI increases both PGC-1α and VEGF mRNA expression in human skeletal muscle when compared to exercise alone. In a second study, we tested the hypothesis that resting CWI (i.e. without the prior stress of exercise) was sufficient to also mediate the activation of such molecular pathways. We provide novel data by demonstrating that superimposing the stress of post-exercise CWI on the prior stress of exercise further increases the expression of both PGC-1α and VEGF mRNA expression in human skeletal muscle. Furthermore, CWI under resting conditions also mediates increased activation of these molecular pathways.

PGC-1α was originally discovered as a cold-inducible transcription co-activator of the adaptive thermogenesis response to environmental conditions such as cold exposure (Puigserver et al. 1998; Cannon et al. 1998), with animal studies confirming chronic whole body cooling increases PGC-1α mRNA (Puigserver et al. 1998; Oliveira et al. 2004). Recently in humans, studies have also shown that acute cold stimuli, as mediated via cold ambient temperatures (Slivka et al. 2013) or CWI (Ihsan et al. 2014), also up-regulates PGC-1α expression. In their recent study, Ihsan et al. (2014), using single-leg CWI (10 °C for 15 min) where the non-immersed leg acted as a control, showed that CWI induced a ~fourfold increase PGC-1α mRNA expression. However, these researchers observed that the non-immersed control leg failed to induce PGC-1α mRNA expression, as would be expected after an exercise stimulus of such duration and intensity. It is difficult to reconcile differences between studies though as differences in muscle fibre recruitment patterns of the vastus lateralis muscle between running and cycling may, in part, be a contributing factor. Consequently, it remained unclear as to whether superimposing the stress of cooling on the prior stress of exercise represents a superior stimulus.

In the present Study 1, the acute bout of high-intensity interval running alone induced a ~fourfold increase in PGC-1α mRNA expression. Moreover, for the first time we report that post-exercise CWI further increased this response (~6-fold) at 3 h post-exercise when compared to exercise alone. As such, these data suggest that acute cold exposure may enhance the response over and above the stress of exercise alone for which to induce the classical hallmark adaptations to endurance training. Indeed Ihsan et al. (2015) recently reported that regular CWI (3 times week^−1^ for 4 weeks) was able to influence the PGC-1α signalling pathway through increased p38 MAPK and AMPK total protein content and phosphorylation. Interestingly, Slivka et al. (2013) recently showed that recovery undertaken in a cold environment (7 °C) for 1 h increased PGC-1α mRNA expression ~eightfold compared with the ~sixfold increase presently noted. These differences likely reflect differences in experimental designs including the type and intensity of exercise (1 h cycling vs. intermittent running), temperature, duration and type of cold stimulus (4 h at 7 °C room temperature vs. 10 min in 8 °C water), differences in populations used and variations in the determination of gene expression. Specifically, differences in the nature of the cooling stimulus (i.e. cold ambient temperature vs. cold water immersion) may be important since the magnitude of sympathetic discharge to skeletal muscle is influenced by both the size of the tissue area exposed to cooling (Seals 1990) and the magnitude of the cooling stimulus (Kregel et al. 1992). Moreover, cold water immersion is likely to offer a greater cooling stimulus via convective heat transfer. Future research is needed to examine the influence of the magnitude of cooling on PGC-1α mRNA expression in human skeletal muscle.

In an attempt to further examine the isolated role of cooling we investigated the impact of CWI at rest. Data from Ihsan et al. (2014) seem to show the significant increase in PGC-1α to largely be a factor of the cold stimulus, primarily due to a lack of increase seen in the exercised control leg. However, despite the profound interest surrounding CWI and the impact it may have on the subsequent adaptive signalling processes since the seminal work by Puigserver et al. (1998), Study 2 is the first to examine the effect of passive CWI on skeletal muscle PGC-1α expression in humans. We presently observed a 1.3-fold increase in PGC-1α mRNA at 3 h after immersion which remained elevated at 6 h (1.4-fold). These findings are consistent with previous observations in animal skeletal muscle which demonstrated that cold exposure, albeit chronic (4 °C for 4 days), promoted a 5.3-fold increase in PGC-1α mRNA expression (Oliveira et al. 2004).

Exposure to a cold environment causes also increase in capillary density in animals (Sillau et al. 1980; Suzuki et al. 1997) and human skeletal muscle (Bae et al. 2003), an effect thought to be mediated through the regulatory role of PGC-1α in facilitating the key pro-angiogenic factor in skeletal muscle, VEGF (Chinsonboom et al. 2009). In animals, Kim et al. (2005) demonstrated that prolonged CWI (1 h day^−1^, 5 days week^−1^ for 20 weeks) evoked higher expressions of VEGF_165_ mRNA and protein when compared to a control. In contrast, in humans, Ihsan et al. (2014), failed to observe any effect of CWI on VEGF mRNA despite apparent activation of PGC-1α. In Study 1, we report that exercise alone does not induce changes in VEGF mRNA expression. In contrast, a significant increase in VEGF_total_ mRNA expression (~2.5 fold) was observed in the CWI condition 3 h post-exercise, with a trend seen for VEGF_165_ (increase ~2.4-fold) thus suggesting a role for cooling. Indeed, the findings in Study 2 demonstrate that CWI alone increases VEGF_165_ mRNA expression by ~1.9- and 2.2-fold at 3 and 6 h post-immersion respectively. To the authors’ knowledge, the present findings are the first to demonstrate increased VEGF mRNA expression in human skeletal muscle in response to acute CWI. These observations are consistent with previous observations indicating that CWI increased VEGF_165_ mRNA approximately ~1.6-fold in rat soleus muscle (Kim et al. 2005). However, further work is required to determine whether regular post-exercise CWI promotes changes in protein content consistent with vascular adaptation to exercise training.

The mechanisms responsible for the cold induction in PGC-1α and VEGF in humans are currently unknown though β-adrenergic activity may influence PGC-1α expression through a number of signalling cascades (Miura et al. 2007). Increased intracellular cAMP and activation and phosphorylation of PKA can result in the binding of CREB (cAMP response element binding protein) to the PGC-1α promoter region, therefore directly influencing its expression (Wu et al. 1999; Puigserver and Spiegelman 2003; Fernandez-Marcos and Auwerx 2011). Parallel to this, β-adrenergic stimulation could impact VEGF expression via PGC-1α and estrogen related receptor α (ERRα) (Chinsonboom et al. 2009). We have previously shown increases in nor-adrenaline following high-intensity exercise are maintained to a greater extent after CWI versus a control, with levels being significantly higher in the CWI condition at 4 h post-exercise (Gregson et al. 2013). In Study 2, CWI elicited a marked increase in nor-adrenaline at 3 h (~54 %) and 6 h (~69 %) after immersion. These findings confirm previous observations where a marked increase (~530 %) in nor-adrenaline concentration was detected following 60 min of CWI (14 °C) (Sramek et al. 2000). Recent observations from our laboratory indicate that increases in nor-adrenaline concentrations following CWI may be more dependent on reduced skin temperature rather than core temperature (Gregson et al. 2013). The decline in skin temperature provokes a profound reflex increase in sympathetic nerve activity through activation of nonnoxious thermoreceptors (Hensel and Boman 1960). Indeed, results from Study 2 showed a reduction in thigh skin temperature of up to 17 °C immediately post-immersion, remaining lower than baseline values until 6 h post-immersion.

A key argument in the case for single-leg immersion protocols in previous research is the attempt of avoiding shivering thermogenesis (Ihsan et al. 2014), which has the ability to influence metabolic and mitochondrial adaptation through calcium and AMPK dependent pathways. As results from the present studies indicate, post-exercise CWI is able to increase the expression in mRNA of these key mitochondrial and angiogenic genes to a greater extent than exercise or passive cooling alone. Additionally, both of the current studies show that, whilst there was an initial cold-shock response as evidenced through significant rises in HR, $\dot{V}{\text{O}}_{2}$ and subjective measures of shivering during the immersion protocol itself, these results returned to baseline (or less) in the immediate recovery period (up to 30 min post-immersion). Whereas it would be naïve to suggest this indicates CWI did not lead to sustained increases in metabolic variables and shivering thermogenesis it is sensible to acknowledge that it is inevitably possible. However, due to the acute nature of such increases seen in metabolic measures we believe the increases in mitochondrial and angiogenic molecular signals seen herein are more temperature driven than a result of shivering thermogenesis.

Limitations to the overall aims were present. A limitation of Study 2 was the failure to use a control condition, with baseline measures serving as the control. However, the authors suggest the increase in PGC-1α mRNA and VEGF165 mRNA predominately reflects cooling rather than any increased mediated sympathetic stress from repeated biopsies. Indeed previous findings have indicated that repeat muscle biopsy sampling at separate sites does not increase VEGF (Gavin et al. 2004) and PGC-1α mRNA (Cartoni et al. 2005). In Study 1 we failed to measure the catecholamine response, however we have previously reported the catecholamine response to this level of post-exercise cooling (Gregson et al. 2013) and the addition of results from Study 2 further demonstrates β-adrenergic stimulation is cold-inducible. Furthermore, it would be sensible to suggest such a response would occur in Study 1 considering the reduction in muscle temperature (3 cm) was greater following post-exercise CWI versus passive (peak reduction of ~5 and 3.5 °C, respectively).

To conclude, the present studies indicate that CWI when used as a post-exercise recovery modality, leads to greater expression of key mitochondrial (PGC-1α) and angiogenic (VEGF) genes than exercise alone therefore modulating the transcriptional adaptation towards a more oxidative phenotype. In addition, passive CWI has the ability to increase the expression of these genes, suggesting possible therapeutic benefits. These results relate well to the practical setting allowing for more informed periodization of recovery strategies to enhance the molecular signalling associated with high-intensity exercise and/or CWI.

## Abbreviations

AMPK: 5′ AMP-activated protein kinase
CONT: Control
CREB: cAMP response element binding protein
CWI: Cold water immersion
ERRα: Estrogen related receptor α
HR: Heart rate
p38 MAPK: p38 mitogen-activated protein kinase
PGC-1α: Peroxisome proliferator activated-receptor γ co-activator-1 α
RER: Respiratory exchange ratio
RPE: Ratings of perceived exertion
VEGF: Vascular endothelial growth factor
$\dot{V}{\text{O}}_{2} \hbox{max}$: Rate of maximal oxygen uptake
